# *catena*-Poly[(chloridozinc)-μ-5-(1-methyl-1*H*-benzimidazol-2-yl-κ*N* ^3^)-1,2,3-triazol-1-ido-κ^2^ *N* ^1^:*N* ^3^]

**DOI:** 10.1107/S1600536812012706

**Published:** 2012-03-31

**Authors:** Chen-Guang Sun, Ji-Rong Song

**Affiliations:** a School of Chemical Engineering, Northwest University, Xi’an 710069, Shaanxi, People’s Republic of China

## Abstract

In the title complex, [Zn(C_10_H_8_N_5_)Cl]_*n*_, the Zn^II^ ion is four-coordinated by one Cl atom and three N atoms from two *in situ*-generated deprotonated 5-(1-methyl-1*H*-benzimidazol-2-yl-κ*N* ^3^)-1,2,3-triazol-1-ide ligands in a slightly distorted tetra­hedral geometry. The Zn^II^ ions are bridged by the ligands, forming a helical chain along [001]. C—H⋯N and C—H⋯Cl hydrogen bonds and π–π inter­actions between the imidazole rings [centroid–centroid distance = 3.4244 (10) Å] assemble the chains into a three-dimensional supra­molecular network.

## Related literature

For general background to hydro­thermal *in situ* reactions, see: Chen & Tong (2007 ▶); Zheng *et al.* (2009 ▶).
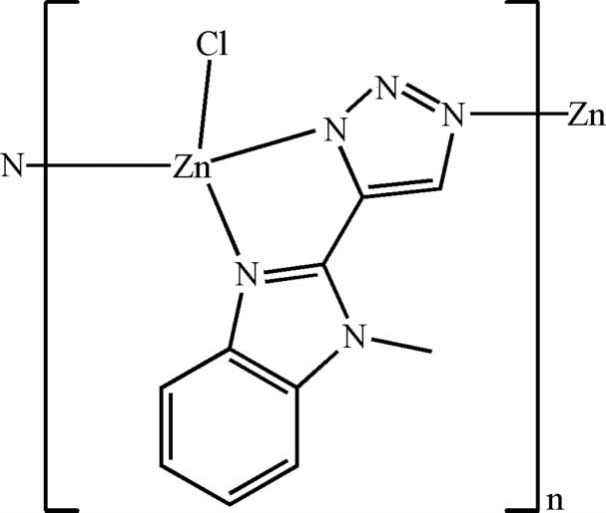


## Experimental

### 

#### Crystal data

[Zn(C_10_H_8_N_5_)Cl]
*M* *_r_* = 299.03
Tetragonal, 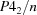

*a* = 16.0822 (1) Å
*c* = 9.0114 (2) Å
*V* = 2330.68 (6) Å^3^
*Z* = 8
Mo *K*α radiation
μ = 2.32 mm^−1^
*T* = 153 K
0.25 × 0.20 × 0.10 mm

#### Data collection

Bruker APEXII CCD diffractometer
Absorption correction: multi-scan (*SADABS*; Bruker, 2001 ▶) *T*_min_ = 0.595, *T*_max_ = 0.801
6487 measured reflections
2511 independent reflections
2242 reflections with *I* > 2σ(*I*)
*R*_int_ = 0.022

#### Refinement

*R*[*F* ^2^ > 2σ(*F* ^2^)] = 0.024
*wR*(*F* ^2^) = 0.061
*S* = 1.05
2511 reflections
155 parameters
H-atom parameters constrained
Δρ_max_ = 0.28 e Å^−3^
Δρ_min_ = −0.32 e Å^−3^

### 

Data collection: *APEX2* (Bruker, 2007 ▶); cell refinement: *SAINT* (Bruker, 2007 ▶); data reduction: *SAINT*; program(s) used to solve structure: *SHELXS97* (Sheldrick, 2008 ▶); program(s) used to refine structure: *SHELXL97* (Sheldrick, 2008 ▶); molecular graphics: *DIAMOND* (Brandenburg, 1999 ▶); software used to prepare material for publication: *SHELXTL* (Sheldrick, 2008 ▶).

## Supplementary Material

Crystal structure: contains datablock(s) I, glogal. DOI: 10.1107/S1600536812012706/hy2526sup1.cif

Structure factors: contains datablock(s) I. DOI: 10.1107/S1600536812012706/hy2526Isup2.hkl

Additional supplementary materials:  crystallographic information; 3D view; checkCIF report

## Figures and Tables

**Table 1:** Hydrogen-bond geometry (Å, °)

*D*—H⋯*A*	*D*—H	H⋯*A*	*D*⋯*A*	*D*—H⋯*A*
C1—H1⋯N5^i^	0.93	2.49	3.274 (2)	142
C10—H10*B*⋯Cl1^ii^	0.96	2.81	3.744 (2)	165

Symmetry codes: (i) 
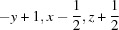
; (ii) 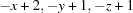.

**Fig. 1.**
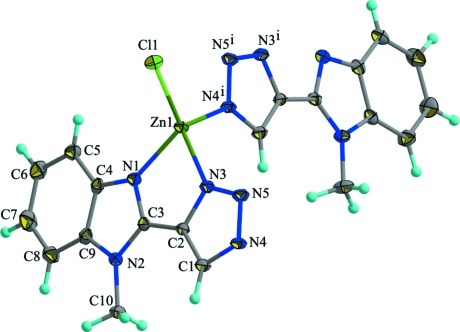
The asymmetric unit of the title complex. Displacement ellipsoids are shown at the 50% probability level. [Symmetry code: (i) 1-y, -1/2+x, -1/2+z.]

**Fig. 2.**
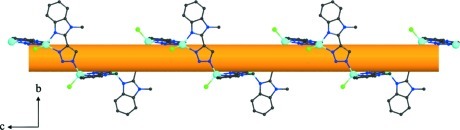
The helical chain in the title complex along the c axis.

**Fig. 3.**
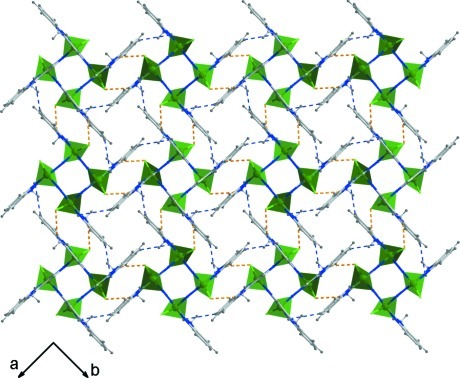
The three-dimensional supramolecular structure connected by hydrogen bonds (C—H···N: blue dashed lines; C—H···Cl: yellow dashed lines).

## supplementary crystallographic information

### Comment

Hydro(solvo)thermal *in situ* metal-ligand reactions, as a new bridge between coordination chemistry and organic synthetic chemistry, with advantages over conventional synthetic routes have attracted intensive interest in recent years (Chen & Tong, 2007). Some hydrothermal *in situ* metal-ligand decarboxylation reactions have been reported in the past (Zheng *et al.*, 2009). According to our research, we found the 5-(1-methyl-1*H*-benzo[*d*]imidazol-2-yl)-3*H*-1,2,3-triazole- 4-carboxylic acid ligand is unstable, when the reaction temperature is high at 160°C. The title compound was obtained by *in situ* decarboxylation reaction. The Zn^II^ atom in the title compound is coordinated by one Cl atom and three N atoms from two deprotonated ligands in a distorted tetrahedral geometry (Fig. 1). The ligands bridge Zn atoms in a µ-κ^3^N,N':N' mode, forming a helical chain structure (Fig. 2). C—H···N and C—H···Cl hydrogen bonds (Table 1) and π–π interactions between the imidazole rings [centroid–centroid distance = 3.4244 (10) Å] assemble the chains into a three-dimensional supramolecular network (Fig. 3).

### Experimental

ZnCl_2_ (0.5 mmol), 5-(1-methyl-1*H*-benzo[*d*]imidazol-2-yl)-3*H*- 1,2,3-triazole-4-carboxylic acid (0.25 mmol) and water (9 ml) were placed in a 15 ml Teflon-lined autoclave. The autoclave was heated at 433 K for 48 h. After the autoclave was cooled to room temperature, yellow block crystals were obtained (yield: *ca* 41.2% based on ligand). The initial ligand 5-(1-methyl-1*H*-benzo[*d*]imidazol-2-yl)-3*H*-1,2,3-triazole- 4-carboxylic acid was sythesized by refluxing *N*-methyl-1,2-benzenediamine dihydrochloride and 1*H*-1,2,3-triazole-4,5-dicarboxylic acid in a 1:1 ratio in HCl (4 mol/L).

### Refinement

H atoms were positioned geometrically and refined as riding atoms, with C—H = 0.93 (CH) and 0.96 (CH_3_) Å and with *U*_iso_(H) = 1.2(1.5 for methyl)*U*_eq_(C).

### Crystal data

**Table:**

[Zn(C_10_H_8_N_5_)Cl]	*D*_x_ = 1.704 Mg m^−^^3^
*M**_r_* = 299.03	Melting point: 178 K
Tetragonal, *P*4_2_/*n*	Mo *K*α radiation, λ = 0.71073 Å
Hall symbol: -P 4bc	Cell parameters from 3164 reflections
*a* = 16.0822 (1) Å	θ = 2.8–27.6°
*c* = 9.0114 (2) Å	µ = 2.32 mm^−^^1^
*V* = 2330.68 (6) Å^3^	*T* = 153 K
*Z* = 8	Block, yellow
*F*(000) = 1200	0.25 × 0.20 × 0.10 mm

### Data collection

**Table:**

Bruker APEXII CCD diffractometer	2511 independent reflections
Radiation source: fine-focus sealed tube	2242 reflections with *I* > 2σ(*I*)
Graphite monochromator	*R*_int_ = 0.022
φ and ω scans	θ_max_ = 27.0°, θ_min_ = 2.9°
Absorption correction: multi-scan (*SADABS*; Bruker, 2001)	*h* = −20→19
*T*_min_ = 0.595, *T*_max_ = 0.801	*k* = −20→11
6487 measured reflections	*l* = −11→9

### Refinement

**Table:**

Refinement on *F*^2^	Primary atom site location: structure-invariant direct methods
Least-squares matrix: full	Secondary atom site location: difference Fourier map
*R*[*F*^2^ > 2σ(*F*^2^)] = 0.024	Hydrogen site location: inferred from neighbouring sites
*wR*(*F*^2^) = 0.061	H-atom parameters constrained
*S* = 1.05	*w* = 1/[σ^2^(*F*_o_^2^) + (0.0248*P*)^2^ + 1.2538*P*] where *P* = (*F*_o_^2^ + 2*F*_c_^2^)/3
2511 reflections	(Δ/σ)_max_ = 0.001
155 parameters	Δρ_max_ = 0.28 e Å^−^^3^
0 restraints	Δρ_min_ = −0.32 e Å^−^^3^

### Special details

**Table:**

Geometry. All e.s.d.'s (except the e.s.d. in the dihedral angle between two l.s. planes) are estimated using the full covariance matrix. The cell e.s.d.'s are taken into account individually in the estimation of e.s.d.'s in distances, angles and torsion angles; correlations between e.s.d.'s in cell parameters are only used when they are defined by crystal symmetry. An approximate (isotropic) treatment of cell e.s.d.'s is used for estimating e.s.d.'s involving l.s. planes.
Refinement. Refinement of *F*^2^ against ALL reflections. The weighted *R*-factor *wR* and goodness of fit *S* are based on *F*^2^, conventional *R*-factors *R* are based on *F*, with *F* set to zero for negative *F*^2^. The threshold expression of *F*^2^ > σ(*F*^2^) is used only for calculating *R*-factors(gt) *etc*. and is not relevant to the choice of reflections for refinement. *R*-factors based on *F*^2^ are statistically about twice as large as those based on *F*, and *R*- factors based on ALL data will be even larger.

### Fractional atomic coordinates and isotropic or equivalent isotropic displacement parameters (Å^2^)

**Table:**

	*x*	*y*	*z*	*U*_iso_*/*U*_eq_	
Zn1	0.885173 (13)	0.359714 (13)	0.23661 (2)	0.01485 (8)	
Cl1	0.98564 (3)	0.32498 (3)	0.08256 (5)	0.02279 (12)	
N4	0.86431 (9)	0.22361 (10)	0.63125 (16)	0.0164 (3)	
N1	0.89656 (9)	0.46000 (9)	0.37457 (16)	0.0150 (3)	
N3	0.87375 (10)	0.29967 (9)	0.43575 (16)	0.0158 (3)	
C3	0.88790 (11)	0.43874 (11)	0.51738 (19)	0.0143 (4)	
N2	0.88891 (9)	0.50546 (9)	0.60836 (16)	0.0156 (3)	
N5	0.86571 (10)	0.22235 (10)	0.48211 (16)	0.0174 (3)	
C9	0.89630 (11)	0.57556 (11)	0.5191 (2)	0.0171 (4)	
C10	0.87849 (13)	0.50517 (12)	0.7694 (2)	0.0224 (4)	
H10A	0.8203	0.5044	0.7931	0.034*	
H10B	0.9035	0.5542	0.8106	0.034*	
H10C	0.9048	0.4567	0.8102	0.034*	
C2	0.87783 (11)	0.35173 (11)	0.5545 (2)	0.0146 (4)	
C4	0.90207 (11)	0.54619 (11)	0.3728 (2)	0.0164 (4)	
C8	0.89762 (13)	0.65991 (12)	0.5530 (2)	0.0242 (4)	
H8	0.8937	0.6790	0.6501	0.029*	
C1	0.87145 (12)	0.30313 (11)	0.6796 (2)	0.0175 (4)	
H1	0.8719	0.3212	0.7776	0.021*	
C7	0.90506 (14)	0.71385 (13)	0.4342 (2)	0.0294 (5)	
H7	0.9049	0.7708	0.4519	0.035*	
C6	0.91287 (14)	0.68529 (13)	0.2877 (2)	0.0269 (5)	
H6	0.9187	0.7237	0.2113	0.032*	
C5	0.91205 (12)	0.60159 (12)	0.2543 (2)	0.0207 (4)	
H5	0.9179	0.5828	0.1573	0.025*	

### Atomic displacement parameters (Å^2^)

**Table:**

	*U*^11^	*U*^22^	*U*^33^	*U*^12^	*U*^13^	*U*^23^
Zn1	0.01681 (12)	0.01827 (12)	0.00945 (12)	−0.00095 (8)	−0.00090 (8)	0.00094 (8)
Cl1	0.0229 (2)	0.0266 (2)	0.0189 (2)	−0.0002 (2)	0.00538 (18)	−0.00207 (19)
N4	0.0217 (8)	0.0182 (8)	0.0093 (7)	0.0009 (7)	0.0008 (6)	0.0005 (6)
N1	0.0151 (7)	0.0163 (7)	0.0136 (7)	−0.0006 (6)	−0.0009 (6)	0.0009 (6)
N3	0.0202 (8)	0.0158 (8)	0.0115 (7)	−0.0012 (6)	−0.0002 (6)	0.0006 (6)
C3	0.0120 (8)	0.0170 (9)	0.0139 (8)	−0.0010 (7)	−0.0004 (7)	−0.0008 (7)
N2	0.0168 (7)	0.0164 (7)	0.0137 (8)	−0.0026 (6)	−0.0004 (6)	−0.0006 (6)
N5	0.0232 (8)	0.0181 (8)	0.0108 (7)	−0.0002 (7)	−0.0002 (6)	0.0029 (6)
C9	0.0143 (9)	0.0185 (9)	0.0185 (9)	−0.0004 (7)	−0.0013 (7)	0.0023 (7)
C10	0.0319 (11)	0.0227 (10)	0.0126 (9)	−0.0054 (9)	0.0021 (8)	−0.0028 (7)
C2	0.0142 (8)	0.0177 (9)	0.0119 (9)	0.0005 (7)	−0.0002 (7)	−0.0022 (7)
C4	0.0127 (8)	0.0177 (9)	0.0187 (9)	−0.0018 (7)	−0.0018 (7)	0.0013 (7)
C8	0.0301 (11)	0.0202 (10)	0.0222 (10)	−0.0013 (9)	−0.0004 (9)	−0.0028 (8)
C1	0.0222 (9)	0.0181 (9)	0.0121 (8)	0.0004 (8)	0.0011 (7)	−0.0018 (7)
C7	0.0396 (13)	0.0161 (10)	0.0325 (12)	−0.0029 (9)	−0.0025 (10)	0.0010 (9)
C6	0.0338 (12)	0.0214 (10)	0.0253 (11)	−0.0045 (9)	−0.0019 (9)	0.0084 (9)
C5	0.0210 (10)	0.0228 (10)	0.0184 (10)	−0.0022 (8)	−0.0020 (8)	0.0040 (8)

### Geometric parameters (Å, º)

**Table:**

Zn1—N4^i^	1.9920 (15)	C9—C8	1.391 (3)
Zn1—N1	2.0445 (15)	C9—C4	1.404 (3)
Zn1—N3	2.0461 (15)	C10—H10A	0.9600
Zn1—Cl1	2.2022 (5)	C10—H10B	0.9600
N4—N5	1.344 (2)	C10—H10C	0.9600
N4—C1	1.356 (2)	C2—C1	1.376 (3)
N4—Zn1^ii^	1.9920 (15)	C4—C5	1.400 (3)
N1—C3	1.339 (2)	C8—C7	1.383 (3)
N1—C4	1.389 (2)	C8—H8	0.9300
N3—N5	1.318 (2)	C1—H1	0.9300
N3—C2	1.360 (2)	C7—C6	1.403 (3)
C3—N2	1.351 (2)	C7—H7	0.9300
C3—C2	1.448 (2)	C6—C5	1.380 (3)
N2—C9	1.390 (2)	C6—H6	0.9300
N2—C10	1.461 (2)	C5—H5	0.9300
			
N4^i^—Zn1—N1	109.83 (6)	N2—C10—H10B	109.5
N4^i^—Zn1—N3	110.90 (6)	H10A—C10—H10B	109.5
N1—Zn1—N3	81.20 (6)	N2—C10—H10C	109.5
N4^i^—Zn1—Cl1	110.69 (5)	H10A—C10—H10C	109.5
N1—Zn1—Cl1	121.17 (4)	H10B—C10—H10C	109.5
N3—Zn1—Cl1	119.94 (5)	N3—C2—C1	106.92 (16)
N5—N4—C1	109.51 (15)	N3—C2—C3	114.77 (16)
N5—N4—Zn1^ii^	117.63 (12)	C1—C2—C3	138.31 (17)
C1—N4—Zn1^ii^	132.73 (12)	N1—C4—C5	130.65 (18)
C3—N1—C4	105.82 (15)	N1—C4—C9	108.71 (16)
C3—N1—Zn1	111.95 (12)	C5—C4—C9	120.64 (17)
C4—N1—Zn1	141.79 (12)	C7—C8—C9	116.31 (18)
N5—N3—C2	109.65 (14)	C7—C8—H8	121.8
N5—N3—Zn1	137.05 (12)	C9—C8—H8	121.8
C2—N3—Zn1	113.26 (12)	N4—C1—C2	106.21 (16)
N1—C3—N2	112.29 (16)	N4—C1—H1	126.9
N1—C3—C2	118.70 (16)	C2—C1—H1	126.9
N2—C3—C2	128.99 (16)	C8—C7—C6	122.04 (19)
C3—N2—C9	107.11 (15)	C8—C7—H7	119.0
C3—N2—C10	126.80 (16)	C6—C7—H7	119.0
C9—N2—C10	125.97 (16)	C5—C6—C7	121.60 (19)
N3—N5—N4	107.71 (14)	C5—C6—H6	119.2
N2—C9—C8	131.72 (17)	C7—C6—H6	119.2
N2—C9—C4	106.03 (16)	C6—C5—C4	117.12 (18)
C8—C9—C4	122.24 (17)	C6—C5—H5	121.4
N2—C10—H10A	109.5	C4—C5—H5	121.4

Symmetry codes: (i) −*y*+1, *x*−1/2, *z*−1/2; (ii) *y*+1/2, −*x*+1, *z*+1/2.

### Hydrogen-bond geometry (Å, º)

**Table:**

*D*—H···*A*	*D*—H	H···*A*	*D*···*A*	*D*—H···*A*
C1—H1···N5^iii^	0.93	2.49	3.274 (2)	142
C10—H10*B*···Cl1^iv^	0.96	2.81	3.744 (2)	165

Symmetry codes: (iii) −*y*+1, *x*−1/2, *z*+1/2; (iv) −*x*+2, −*y*+1, −*z*+1.
